# Is there any Relationship Between C-Reactive Protein Level and Complex Coronary Plaques in Patients with Unstable Angina?

**Published:** 2010

**Authors:** Masoumeh Sadeghi, Masoud Pourmoghaddas, Aliakbar Tavasoli, Hamidreza Roohafza

**Affiliations:** 1 Associate Professor, Isfahan Cardiovascular Research Center, Isfahan University of Medical Sciences, Isfahan, Iran; 2 Professor, Isfahan University of Medical Sciences, Isfahan, Iran; 3 Associate Professor, Isfahan University of Medical Sciences, Isfahan, Iran; 4 Psychiatrist, Isfahan Cardiovascular Research Center, Isfahan University of Medical Sciences, Isfahan, Iran

**Keywords:** CRP, Simple and complex lesion, Unstable angina, Angiography

## Abstract

**BACKGROUND:**

Coronary artery disease is a leading cause of death in developed countries. On the other hand, increased level of CRP has been seen in atherosclerosis. According to this finding, we decided to conduct this study to assess the relationship between CRP and complex lesions of coronary arteries in patients with unstable angina.

**METHODS:**

In this analytical cross sectional study which was conducted in 2007 in Chamran hospital, samples were collected using simple sampling and included 80 patients who referred to the hospital due to angina pectoris, had the diagnosis of unstable angina and were candidates of angiography. At first, a questionnaire was filled for each patient including demographic factors and their medical history. Then a blood sample was taken to assess level of CRP, FBS, and lipid profiles. The results of angiographic studies were considered by three cardiologists and abnormal patients were classified into simple and complex groups according to Ambrose criteria. Data was analyzed using SPSS version 15, t-student and chi-square tests.

**RESULTS:**

Mean age of samples was 58.27 ± 6.23 years old. Considering the risk factors, most simple and complex lesions happened in obese patients however the only significant difference was observed in BMI between two groups (P < 0.05). Mean level of CRP in the population under study was 6.05 ± 4 mg/dl which was 1.37 ± 2 and 8.01 ± 6 in simple and complex groups respectively (P < 0.05). CRP mean was significantly higher in the group with complex lesions, less than 1 mg/dl in simple lesion and more than 4 mg/dl in complex lesions.

**CONCLUSION:**

According to our findings, there is a significant difference considering CRP level in unstable angina patients who have complex lesions compared with simples ones. As complex plaques are more susceptible to develop coronary events, patients with a higher probability of complicated lesions can be screened.

## Introduction

Coronary artery disease (CVD) is a leading cause of death worldwide. World Health Organization (WHO) has announced that this disease will be the first life threatening factor in 2020.^1^ One of the main causes of CVD is atherosclerosis which gradually develops from the first decade of life. However its clinical presentations will finally form cerebrovascular accidents, myocardial infarction or even death.^2^ Multiple risk factors will accelerate atherosclerosis including hypertension, diabetes, hyperlipidemia, and smoking.^3^

Role of inflammatory factors has been proposed recently. C-reactive protein (CRP) is among those factors which are produced by liver within 8 to 12 hours after infection or inflammation. Changes in a vessel wall disappear in the plaque location after release of cytokines and after leukocytes have entered and muscular cells migrated. Inflammatory factors including these proteins are released in the blood.^4^ One study conducted on healthy population revealed that CRP is capable of predicting coronary events and its high level implies high risk healthy individuals.^5^

Coronary plaque morphology, according to studies by Ambrose, was divided into two groups of simple and complex. Simple lesions always had thick necks while complex ones had narrow necks with suspending ridges and irregular borders.^6^–^8^

Acute coronary events mostly happen in complex lesions but is there a relationship between inflammation and complex lesions which leads to more frequent coronary events? Multiple studies declare the relationship between these two.^9^, ^10^

In this study, by using CRP measurements which is available anywhere, and evaluation of angiographic morphology of coronary plaques in patients with unstable angina, we can consider correlation of inflammation and complex plaque and then we can relate CRP as an inflammatory marker with high risk unstable patients that need more intensive medical or interventional follow-up.

## Materials and Methods

This cross sectional study was conducted on 80 consecutive patients referred to Chamran hospital due to unstable angina and underwent coronary artery angiography in 2007. Unstable angina is defined as angina at rest with an accelerating pattern (with more frequency, higher intensity or longer duration), rest angina or an angina which has recently developed.^10^

Patients with a history of myocardial infarction, surgery and/or PTCA, valvular disease, anemia, fever, thyroid abnormalities, and renal failure, inflammatory and rheumatologic diseases or patients taking related medications including corticosteroid were excluded from the study. Letters of consent were taken from patients regarding all steps of study.

A questionnaire including information about demographic factors, history of above mentioned diseases, drug consumption, and smoking was filled for those patients who met the inclusion criteria. Then their weight and height were measured and Body Mass Index (BMI) was calculated accordingly.^11^ Patients' systolic and diastolic blood pressures were measured. Hypertension was defined as blood pressure above 140/90 and/or taking hypertensive medications.^12^ After applying a written consent from patients, their venous blood sample was taken to measure high density lipoprotein (HDL-C) and fasting blood sugar (FBS). Diabetes mellitus was defined as having blood sugar equal or more than 126 mg/dl; HDL concentration less than 50 mg/dl in women and less than 40 mg/dl in men was considered as HDL-C abnormality.^13^, ^14^ Moreover, CRP concentration was measured using spectrophotometer.^15^ In the last step, all patients underwent coronary artery angiography according to Judkins standard protocol. The degree of stenosis in each epicardial coronary arteries was evaluated by three cardiologists who were blind about laboratory test results of the patients. Atherothrombotic plaques were classified into simple and complex according to Ambrose criteria.^16^ The study protocol was reviewed and approved by the Isfahan Cardiovascular Research Center Ethics Committee, which is a member of the Office for Human Research Protections, US Department of Health and Human Services. Data were analyzed using SPSS version 15 while independent t test and Chi square were used for comparing mean averages.

## Results

Among all 80 patients with mean age of 58.27 ± 6.23 years old, 40 ones had complex coronary artery lesions, and 49 patients (61.42%) were male. Almost all patients had at least one significant lesion in any epicardial artery.

The population status considering diabetes mellitus, hypertension, low HDL-C, obesity and smoking is shown in table 1. Most complex and simple lesions existed in obese patients. The difference was only significant in coronary plaques of obese patient (P < 0.001), while the difference was not significant with other risk factors.

**Table 1:** Frequency of coronary artery disease risk factors in the population under study

Lesion Risk factor	Simple plaque	Complex plaque	P value

	n (%)	n (%)	
Diabetes mellitus	10 (28.57)	14 (40)	0.31
Hypertension	11 (31.42)	11 (31.42)	1
Low HDL-C	14 (40)	26 (74.28)	0.003
Obesity	10 (28.57)	8 (22.85)	0.5
Smoking			

*P value less than 0.05 is significant

In table 2, results related to the average amount of HDL-C, FBS, BMI, systolic and diastolic blood pressure in two groups with simple and complex lesions were assessed and it was concluded that all averages were higher in the group with complex lesions. However, the relationship was only significant in BMI (P < 0.05). CRP level in simple and complex lesion were 1.37 ± 2 and 8.01 ± 6 respectively (P = 0.005).

**Table 2:** Comparison between the mean and standard deviation of main risk factors of coronary artery disease in the population under study

Mean type of lesion	BMI (kg/m2)	HDL (mg/dl)	FBS (mg/dl)	Diastolic blood pressure (mmHg)	Systolic blood pressure (mmHg)
Simple	23.79 ± 4.08	40.23 ± 10.44	123.51 ± 46.86	124.71 ± 21.24	72.14 ± 11.3
Complex	26.83 ± 3.9	40 ± 8.23	153.94 ± 81.82	120.14 ± 17.42	72.86 ± 10.7
P	0.002	0.92	0.06	0.32	0.78

*P value less than 0.05 is significant

According to table 3, CRP level was divided into three groups, less than 1, between 1 to 4, and more than 4 mg/dl. Most complex lesions were seen in CRP concentrations higher than 4 mg/dl (P = 0.02).

**Table 3:** CRP frequency distribution in the population under study

CRP level (mg/dl) Lesion type	≤ 1	2–3	≥ 4	P value
Simple number (%)	19 (54.3)	10 (28.5)	6 (17.2)	0.02
Complex number (%)	12 (37)	10 (28.5)	12 (34.5)	

According to chi-square analysis

## Discussion

Role of inflammation has been suggested in acute coronary syndrome pathogenesis as plaque rupture may lead to complex lesions and myocardial infarction.^1^, ^17^ On the other hand, CRP may increase atherosclerosis process.

According to Ridkers' study prognostic value of CRP is even higher than cholesterol level while results of a study which was conducted by Kimura revealed that an increase in CRP level following myocardial infarction accompanies less myocardial destruction and better left ventricular function.^18^, ^19^ As a result, defining CRP prognostic value is not clear yet. On the other hand, complex lesions are more probable to produce higher complications.^20^–^22^ In the present study, it was obviously revealed that CRP level is significantly higher in patients with unstable angina and complex lesions. However a significant difference was observed only in BMI comparing two groups of simple and complex lesions. Moreover, obese patients had more complex lesions. Goldstein showed in his study that coronary artery disease risk factors were not significantly different in the groups with simple and complex lesions.^23^

In another study conducted by Zairis, it was declared that there is a direct relationship between CRP and complex plaque. The diffuse inflammatory process throughout the whole coronary artery vessels in acute coronary syndrome may lead to instability in coronary plaques.^24^ Some other studies have shown lacerated plaque and complex in lesions patients with myocardial infarction.^23^, ^25^ These patients had higher levels of CRP.

The direct effect of CRP on endothelial cells through VCAM-1 (Vascular Cell Adhesion Molecule), E-selection and ICAM-1 (IntraCellular Adhesion Molecule) has been declared which could be due to an increase in adhesive molecules presentation in vessel wall. As a result, CRP is not only a sign of general inflammation but is a factor which accelerates atherosclerosis.^4^ Generally, inflammatory cell activity may increase secretion of metalloproteinase or cytokines which lead to degeneration of matrix in atherosclerotic plaques. This could form complex lesions and an increase in CRP production in liver.^24^ According to the present study and other similar studies, it could be concluded that CRP level is significantly higher in complex lesions and probably an aggravated inflammation in the plaques will lead to their instability. In addition, taking this for granted that most coronary events happen in plaques with mild stenosis could declare that inflammation can lead to plaque rupture and acute events.^26^ According to these results, it is recommended to consider CRP level together with other risk predicting factors including clinical presentation and laboratory findings in patients with unstable angina. This could lead to an implementation of more improved treatments for patients with acute coronary syndromes. Hence, it is suggested to conduct a cohort study with more cardiac patients in various age and sex groups to determine the prognostic value of CRP as an inflammatory marker in forming complications in ischemic patients.
